# Evaluation of the Effects of Carbon 60 Nanoparticle Exposure to Adult Zebrafish: A Behavioral and Biochemical Approach to Elucidate the Mechanism of Toxicity

**DOI:** 10.3390/ijms19123853

**Published:** 2018-12-03

**Authors:** Sreeja Sarasamma, Gilbert Audira, Stevhen Juniardi, Bonifasius Putera Sampurna, Yu-Heng Lai, Erwei Hao, Jung-Ren Chen, Chung-Der Hsiao

**Affiliations:** 1 Department of Chemistry, Chung Yuan Christian University, Chung-Li 32023, Taiwan; sreejakarthik@hotmail.com (S.S.); gilbertaudira@yahoo.com (G.A.); 2 Department of Bioscience Technology, Chung Yuan Christian University, Chung-Li 32023, Taiwan; stvn.jun@gmail.com (S.J.); boni_bt123@hotmail.com (B.P.S.); 3 Department of Chemistry, Chinese Culture University, Taipei 11114, Taiwan; LYH21@ulive.pccu.edu.tw; 4 Guangxi Key Laboratory of Efficacy Study on Chinese Materia Medica, Guangxi University of Chinese Medicine, Nanning 530200, China; 5 Guangxi Collaborative Innovation Center for Research on Functional Ingredients of Agricultural Residues, Guangxi University of Chinese Medicine, Nanning 530200, China; 6 Department of Biological Science & Technology College of Medicine, I-Shou University, Kaohsiung, 82445, Taiwan; 7 Center for Biomedical Technology, Chung Yuan Christian University, Chung-Li 32023, Taiwan; 8 Center for Nanotechnology, Chung Yuan Christian University, Chung-Li 32023, Taiwan

**Keywords:** zebrafish, behavior, fullerene, hypoactivity, anxiety, ecotoxicity

## Abstract

There is a growing concern for the potential toxicity of engineered nanomaterials that have made their way into virtually all novel applications in the electronics, healthcare, cosmetics, technology, and engineering industries, and in particular, biomedical products. However, the potential toxicity of carbon 60 (C_60_) at the behavioral level has not been properly evaluated. In this study, we used idTracker, a multitracking algorithm to quantitatively assess behavioral toxicity induced by C_60_ nanoparticles (C_60_ NPs) in adult zebrafish. We demonstrated that locomotion, novel tank exploration, aggression, shoaling, and color preference activities of the C_60_ NPs-treated fish was significantly reduced. In addition, the C_60_ NPs-treated fish also displayed dysregulation of the circadian rhythm by showing lower locomotion activities in both day and night cycles. The biochemical results showed that C_60_ NPs exposure at low concentration induced oxidative stress and DNA damage, reduced anti-oxidative capacity and ATP (adenosine triphosphate) levels, and induced stress-associated hormones, hypoxia, as well as inflammation marker upregulation in muscle and gill tissues. Together, this work, for the first time, provide direct evidence showing that the chronic exposure of C_60_ NPs induced multiple behavioral abnormalities in adult zebrafish. Our findings suggest that the ecotoxicity of C_60_ NPs towards aquatic vertebrates should be carefully evaluated.

## 1. Introduction

Nanoparticles (NPs) hold a promising future in biomedical applications such as biosensors, target drug/gene delivery, cancer therapy, bio-imaging, and antimicrobial approaches owing to its unique physical, chemical, and mechanical properties besides minimal toxicity to normal cells and photo stability [1,2]. Over the years, a sharp increase in the usage of these materials in various industry and health fields resulted in their copious release into the environment and aquatic biota [3,4]. Carbon 60 nanoparticles (C_60_ NPs) have been used in many commercial processes and industrial products such as IT (intelligent technology) devices and diagnostics, as well as in environmental, pharmaceutical, superconductor, and energy industries [5,6]. Fullerenes are the third pure form of carbon. Since, fullerenes are isolated from soot, which cause cancer and lung disease, and are the second most common human cause of global warming. Whether C_60_ itself is biologically safe is still up for a controversial debate [7,8,9]. Some research results showed that C_60_ itself is biologically inert and can absorb free radicals playing a role as an anti-oxidative reagent in vitro and in vivo [10,11,12]. However, some research results showed that C_60_ administration via inhalation into the air track will cause severe pulmonary toxicity in rats [13,14] (summarized in Supplementary Table S1). Therefore, the potential biological toxicity of C_60_ deserves more attention and more studies should be conducted in different model organisms in order to obtain a full toxicological spectrum of C_60_ NPs. In this consideration, there is a need to decipher bio-behavioral mechanisms through interdisciplinary methods to study the toxicity of the above materials. However, the unique features and bioavailability of C_60_ NPs are brimming with uncertainties and concerns environmental and occupational exposure. Variations in the physiochemical and biological properties of C_60_ NPs affect biological responses including reactive oxygen species (ROS) production [15,16]. Unfortunately, data regarding the impact of C_60_ NPs on the natural environment and human health is very limited.

Aquatic environments are often contaminated by consumer products such as cosmetic products, sun screens, tooth paste, paints, and air blasting media [17,18,19]. The global extent of contamination by engineered nanoparticles is largely unknown, and it is especially difficult to estimate as the number of various products that use nanomaterials is rapidly on the rise. Over the past decades, both zebrafish embryos and adults have served as an excellent model for nanoparticle toxicity assessment [20,21]. Primarily, zebrafish have exceptional tissue transparency at the embryonic stage, making it easy to study potential chemical toxicity at the organ or even whole organism level [22]; Secondly, zebrafish can breed thousands of eggs making it possible to obtain biological duplicates from the same breeding batch, thereby reducing potential individual variations; Thirdly, since zebrafish are an aquatic animal, the exposure of NPs is relative easy in that the nanoparticle can easily penetrate fish egg chorion and can later be absorbed by embryos or adults through water/food uptake [23].

**Figure 1:**
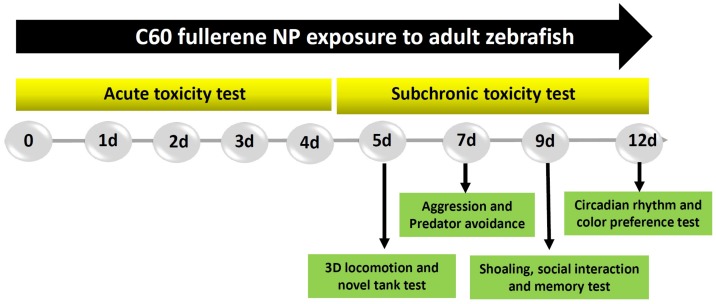
Overview of the experimental design and time points for a sub-chronic toxicity test for carbon 60 nanoparticles (C_60_ NPs) in adult zebrafish. For sub-chronic toxicity, we measured 3D (three-dimensional) locomotion, novel tank exploration, aggression, predator avoidance, social interaction, shoaling, memory, circadian rhythm, and color preference test at specific time points as indicated by the arrowheads. There was no mortality in the whole exposure parameters.

In previous studies, the C_60_ NPs toxicity on aquatic animals has been evaluated in Daphnia and fathead minnow [24]. Recently, potential C_60_ NPs toxicity has been tested by using zebrafish embryos showing potential toxicity to induce pericardial edema, fin malformations, and mortality after exposure at 200–300 ppb [15]. However, the impact of C_60_ NPs on behavioral toxicity for adult zebrafish remains poorly understood. Therefore, in this study, we aimed to explore the potential adverse effect of sub-chronic C_60_ NPs exposure on behavioral profiles of adult zebrafish based on different parameters, namely, locomotion, exploratory test, passive avoidance, predator avoidance, aggression, social interaction, shoaling paradigm, circadian rhythm, and color preference experiments (the experimental design is illustrated in Figure 1). In this study, we used idTracker, a robust and open source tracking algorithm to analyze the behavior of zebrafish [25]. Here, we reported three highlights for C_60_ NPs toxicity in adult zebrafish for the first time: (1) Ours is the first study to report the C_60_ NPs induced changes in behavioral paradigms specifically, and less locomotor co-ordination session in low concentrations of C_60_ NPs exposed in zebrafish; (2) We demonstrated that sub-chronic exposure of sub-lethal concentrations of C_60_ NPs caused reduced aggression and anxiolytic character in C_60_ NPs exposed zebrafish; (3) Finally, we showed that adult zebrafish sub-chronically exposed to 1- and 2-ppm C_60_ NPs had a significant increase in oxidative stress biomarkers such as reactive oxygen species (ROS) and TBARS (thiobarbituric acid reactive substances) and a depletion in catalase activity in gill and muscle.

## 2. Results

### 2.1. Determination of C_60_ NPs Size Distribution

**Figure 2:**
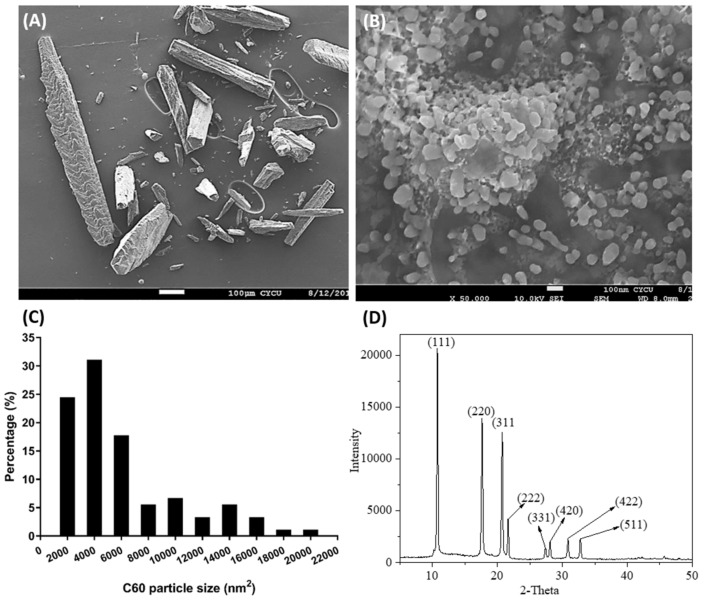
Characterization of the C_60_ NPs used in this study. (**A**) SEM (scanning electron microscope) micrograph of C_60_ NPs stock solution in the absence of solvents; (**B**) C_60_ NPs dissolved in DMSO (dimethyl sulfoxide) showing a wide disparity in aggregation; (**C**) Size distribution of C_60_ NPs dissolved in DMSO; (**D**) X-ray diffraction patterns of the crystal quality of the C_60_ NPs.

Scanning electron microscopy (SEM) and X-ray diffraction (XRD) images of C_60_ NPs at concentrations of 100 mg/L are shown in Figure 2. Particle aggregation was observed for each preparation, resulting in the formation of flocs of different sizes of few hundred nanometers in diameter. Sonication helped to break up larger flocs to smaller discrete aggregates. Without added DMSO (dimethyl sulfoxide), the C_60_ NPs were not dispersed and stayed clearly visible as polymerized strands of approximately 1 µm thick (Figure 2A). Therefore, the addition of DMSO was essential. The SEM analysis showed crystalline and polygonal particles (Figure 2B) a diameter of 54.45 ± 28.34 nm. The crystal structure of C_60_ NPs was characterized by XRD analysis. Figure 2D shows the XRD patterns of the C_60_ NPs. Peaks at 2θ = 11.5°, 17.88°, 23.7°, 24.56°, and 27.45° were assigned to (111), (220), (331), (222), and (331) of C_60_ NPs, respectively. The analysis showed very strong peaks associated with single crystalline C_60_ NPs, which is consistent with the previous report [26].

### 2.2. C_60_ NPs Exposure Reduced Zebrafish Exploration in New Environments and Locomotor Activity

**Figure 3:**
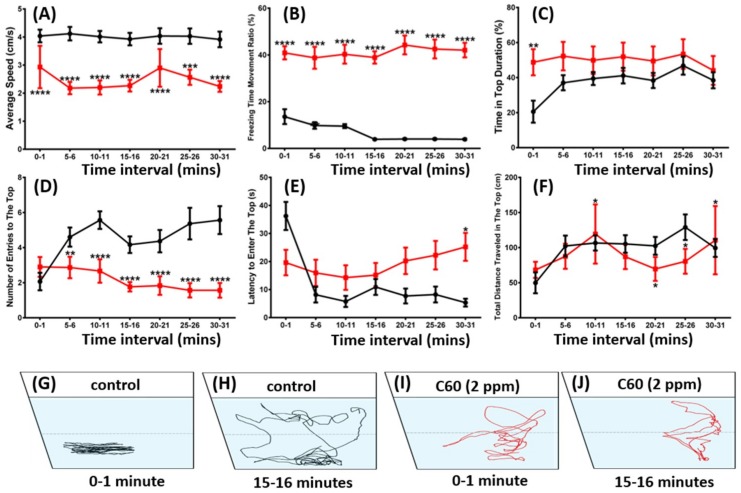
Comparison of behavior endpoints between wild-type and C_60_ NPs-exposed zebrafish in novel tank exploration after five-day exposure. (**A**) Average speed, (**B**) freezing time movement ratio, (**C**) time in top duration, (**D**) number of entries to the top, (**E**) latency to enter the top, and (**F**) total distance traveled in the top were analyzed. The black line represented control and red line represented C_60_ NPs-exposed fish in Figure 3A-F. The swimming trajectories of control (black color in (**G**,**H**)) and C_60_ NPs-exposed fish (red color in (**I**,**J**)) for novel tank exploration test after 1 min and 15 min were recorded and compared. The data are expressed as the mean ± SEM and were analyzed by Mann–Whitney test (*n* = 30 for control; *n* = 30 for C_60_ NPs-exposed fish; * *p* < 0.05, ** *p* < 0.01, *** *p* < 0.005, **** *p* < 0.0001).

The novel tank test is a method to test the fish’s exploration ability in a new environment. When zebrafish are moved into a novel environment, they will display typical bottom dwelling behavior with a high-anxiety level. Later, when fish acclimate to the novel environment, their anxiety level will reduce and gradually move into the upper arena [27]. The novel tank exploration of the adult fish was examined on the 5th day of the C_60_ NPs exposure to determine whether C_60_ NPs can induce any potential acclimation behavior alteration. In contrast to the untreated control fish, we found zebrafish that had been exposed to C_60_ NPs at 2 ppm showed sharp behavioral changes in the novel tank test. Zebrafish exposed to C_60_ NPs displayed reduced average swimming speed (Figure 3A) compared to the control group (*p* < 0.005; *p* < 0.0001). Furthermore, it was found that the number of entries to the top, total distance travelled to the upper zone, and time in top duration (Figure 3C,D,F) also demonstrated that C_60_ NPs affected the exploratory behavior of adult zebrafish (*p* < 0.01; *p* < 0.001, *p* < 0.0001). However, the latency to enter to the top was not altered by the C_60_ NPs (Figure 3E). The novel tank exploration trajectories and video can be found in Figure 3G–J and Supplementary Video S1.

**Figure 4:**
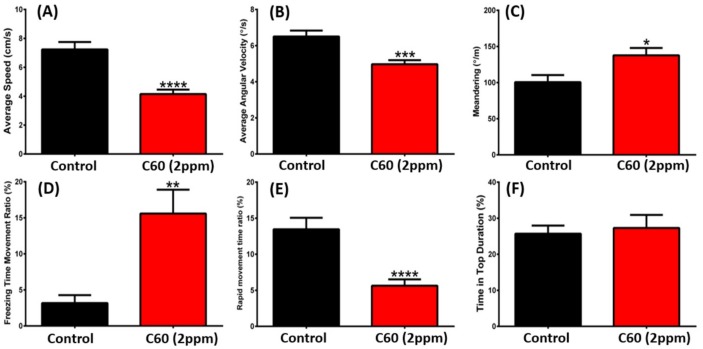
Comparison of behavior endpoints between wild-type and C_60_ NPs-exposed zebrafish in 3D locomotion test after five-day exposure. (**A**) Average speed, (**B**) average angular velocity, (**C**) meandering, (**D**) freezing time movement ratio, (**E**) rapid movement time ratio, and (**F**) time in top duration were analyzed. The data are expressed as the mean ± SEM and were analyzed by *t*-test (*n* = 18 for control; *n* = 30 for C_60_ NPs-exposed fish; * *p* < 0.05, ** *p* < 0.01, *** *p* < 0.005, **** *p* < 0.0001).

**Figure A1:**
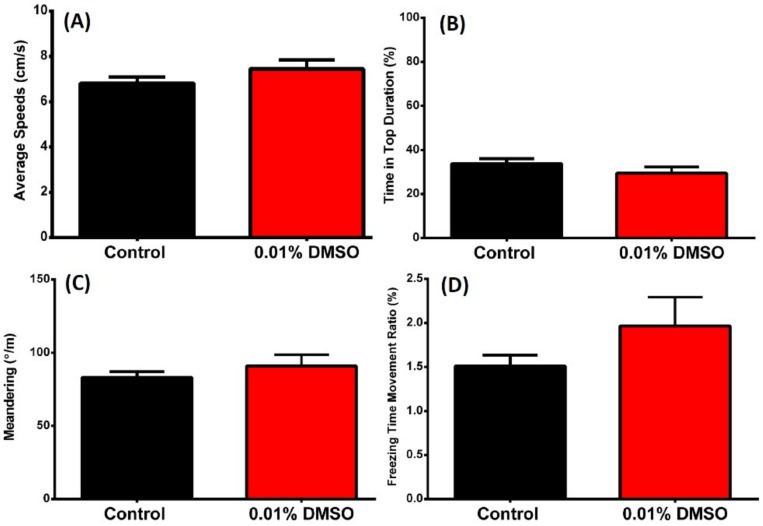
Comparison of behavior endpoints between control and 0.01% DMSO-exposed zebrafish in 3D (three-dimensional) locomotion test after five-day exposure. (A) Average speed, (B) Time in top duration, (C) Meandering, (D) freezing time movement ratio were analyzed. The data are expressed as the mean ± SEM and were analyzed by *t*-test (*n* = 30 for control; *n* = 18 for 0.01% C_60_ NPs-exposed fish).

Similar to the novel tank assay, the 3D locomotion test can track the detailed trajectory in three dimensions and has been reported as a highly-sensitive way to evaluate the potential chemical toxicity at the behavioral level in fish [28,29]. We analyzed fish 3D locomotion activity on 5th day of C_60_ NPs exposure and found that 2-ppm C_60_ NPs exposure resulted in a significant 40% decrease in average speed (Figure 4A) and average angular velocity (Figure 4B) compared to the untreated fish. The freezing time movement ratio was increased in the exposed fish (Figure 4D) compared to the controls (*p* < 0.001). Moreover, it was observed that the adult zebrafish exposed to C_60_ NPs had reduced rapid movement time ratio (Figure 4E) compared with the control group (*p* < 0.0001). In addition, fish exposed to a low concentration of 0.01% DMSO showed a similar level of locomotion activities to those in wild-types (Figure A1). Therefore, the potential detrimental effect of DMSO as a solvent to disperse C_60_ NPs can be ignored.

### 2.3. C_60_ NPs Exposure Reduced Aggression but Not Fear to Predator 

**Figure 5:**
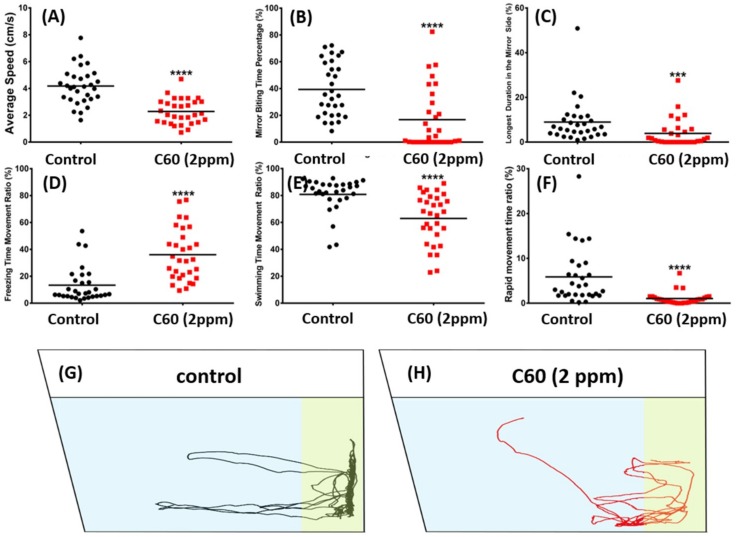
Comparison of mirror biting behavior endpoints between wild-type and C_60_ NPs-exposed zebrafish after seven-day exposure. (**A**) Average speed, (**B**) mirror biting time percentage, (**C**) longest duration in the mirror side, (**D**) freezing time movement ratio, (**E**) swimming time movement ratio, and (**F**) rapid movement time ratio were analyzed. The swimming trajectories of control (black color in (**G**)) and C_60_ NPs-exposed fish (red color in (**H**)) for mirror biting test were recorded and compared. The data are expressed as the means and were analyzed by Mann–Whitney test (*n* = 30 for control; *n* = 30 for C_60_ NPs-exposed fish; *** *p* < 0.001, **** *p* < 0.0001).

Mirror biting assay is a simple and efficient method to test fish aggressiveness in terms of the frequency of the tested fish to bite their mirror images [30]. Mirror biting behavior tests showed that adult zebrafish treated with C_60_ NPs were less aggressive (Figure 5). The C_60_ NPs exposed fish displayed a significant reduction in average swimming speed (Figure 5A), mirror biting time percentage (Figure 5B), and longest duration in the mirror side percentage (Figure 5C) compared to the control group (*p* < 0.001; *p* < 0.0001). Moreover, the C_60_ NPs treated fish showed higher freezing time movement ratio (Figure 5D), lower swimming time movement ratio (Figure 5E; *p* < 0.0001), and lower rapid movement time ratio. The locomotion trajectories and behavioral changes in the aggression test can be found in Figure 5G,H and Supplementary Video S2.

**Figure 6:**
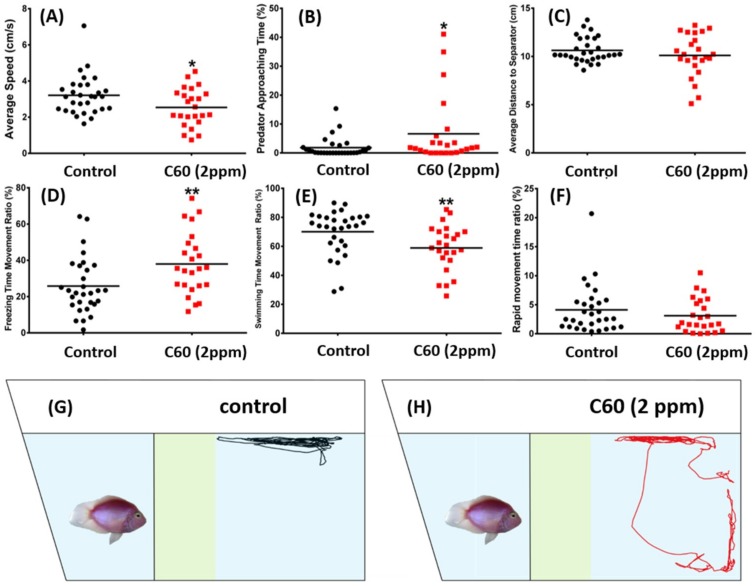
Comparison of predator avoidance behavior endpoints between wild-type and C_60_ NPs-exposed zebrafish after seven-day exposure. (**A**) Average speed, (**B**) predator approaching time percentage, (**C**) average distance to separator, (**D**) freezing time movement ratio, (**E**) swimming time movement ratio, and (**F**) rapid movement time ratio were analyzed. The swimming trajectories of control (black color in (**G**)) and C_60_ NPs-exposed fish (red color in (**H**)) for predator avoidance test were recorded and compared. The data are expressed as the means and were analyzed by Mann–Whitney test (*n* = 30 for control; *n* = 30 for C_60_ NPs-exposured fish; * *p* < 0.05, ** *p* < 0.01).

Predator avoidance is an innate response for fish when facing their natural predator by showing high anxiety or even freezing behavior [31]. We performed a predator avoidance test by incubating zebrafish with predator of convict cichlid (*Amatitlania nigrofasciata*). Six independent measurements were analyzed for predator avoidance test: average speed, predator approaching time percentage, average distance to the separator, freezing time movement ratio, swimming time movement, and rapid movement time ratio. No significant alteration on predator avoidance was found in the C_60_ NPs treated fish. This phenomenon is supported with the same level of average distance to separator between the tested fish and the predator fish observed in the control and the C_60_ NPs treated (Figure 6C), even though a slight increase in predator approaching time was found in the treated group (Figure 6B). This increment was probably caused by high variability of this particular innate response behavior in some treated fish. Unaltered predator avoidance behavior was also characterized by low locomotion activity exhibited by treated fish, which was shown by low average speed and swimming time movement ratio, and high freezing time movement ratio (Figure 6A,D,E). Meanwhile, there was no difference in rapid movement time ratio between the control and the C_60_ NPs treated fish (Figure 6F). The locomotion trajectories and behavioral changes in the predator avoidance test can be found in Figure 6G,H and Supplementary Video S3.

### 2.4. C_60_ NPs Exposure Had No Effect on Social Interaction but Can Reduce Shoaling Behavior

**Figure 7:**
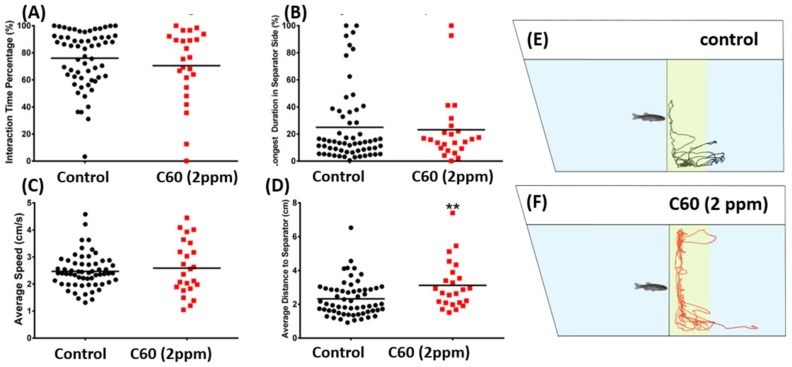
Comparison of conspecific behavior endpoints between wild-type and C_60_ NPs-exposed zebrafish after nine-day exposure. (**A**) Interaction time percentage, (**B**) longest duration in separator side, (**C**) average speed, and (**D**) average distance to separator were analyzed. The swimming trajectories of control (black color in (**E**)) and C_60_ NPs-exposed fish (red color in (**F**)) for social interaction test were recorded and compared. The data are expressed as the means and were analyzed by Mann–Whitney test (*n* = 60 for control; *n* = 24 for C_60_ NPs-exposured fish; ** *p* < 0.01).

Zebrafish are a highly social animal. We tested the potential alteration of zebrafish social interaction after C_60_ NPs exposure by a social interaction test and shoaling test. For social interaction test, we introduced zebrafish into a specially designed tank with a transparent glass separator in the middle. The visiting frequency of two isolated fish at each side was recorded and compared. Results showed there was no significant alteration on social interaction interest between the control and C_60_ NPs exposed animals, in terms of the interaction time percentage (Figure 7A), longest duration on the separator side (Figure 7B), average swimming speed (Figure 7C), and average distance to the separator (Figure 7D). The locomotion trajectories and behavioral changes in the social interaction test can be found in Figure 7E,F and Supplementary Video S4.

**Figure 8:**
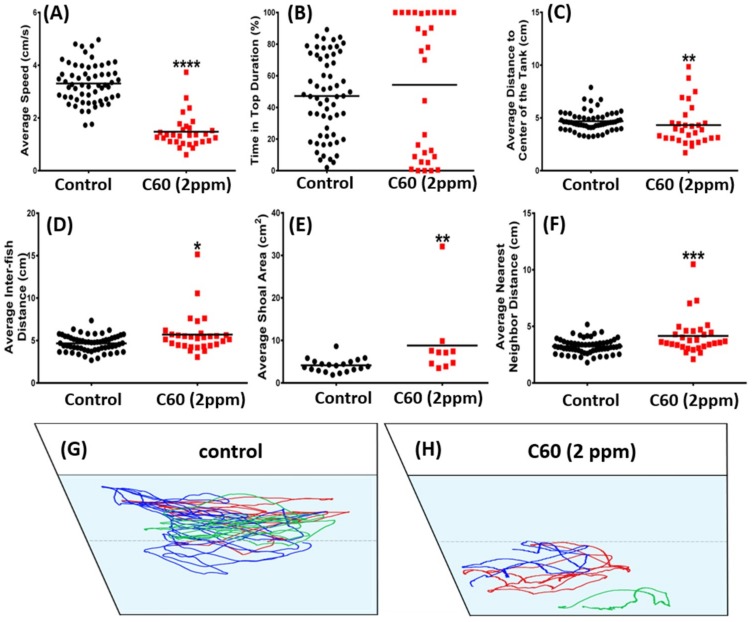
Comparison of shoaling behavior endpoints between wild-type and C_60_ NPs-exposed zebrafish after nine-day exposure. (**A**) Average speed, (**B**) time in top duration, (**C**) average distance to center of the tank, (**D**) average inter-fish distance, (**E**) average shoal area, and (**F**) average nearest neighbor distance were analyzed. The swimming trajectories of the control (**G**) and C_60_ NPs-exposed fish (**H**) for shoaling test were recorded and compared. Three fish were tested for shoaling behavior and their trajectories were labeled by different color for clarifying their identity. The data are expressed as the means and were analyzed by Mann–Whitney test (*n* = 60 for control; *n* = 30 for C_60_ NPs-exposed fish; * *p* < 0.05, ** *p* < 0.01, *** *p* < 0.005, ****, *p* < 0.0001).

Shoaling is an innate behavior for fish to swim together in order to reduce anxiety and the risk being captured by the predators [32,33]. For the shoaling test, six endpoints were assessed in terms of average speed, time on top duration, average distance to center of the tank, average inter-fish distance, average shoal area, average nearest neighbor distance, average farthest neighbor distance, providing overall swimming speed, and reaction to the shoal. There was a significant decrease of average speed, indictive of less locomotion activity (Figure 8A; *p* < 0.0001) in the C_60_ NPs treated group compared to the control group. For average distance to the center of the tank, there was a significant difference in the treated group (Figure 8C; *p* < 0.001). There was also a significant increase on average inter-fish distance (Figure 8D) and shoaling area (Figure 8E) in the C_60_ NPs exposed group. However, the average nearest neighbor distance travelled has significantly increased (Figure 8F; *p* < 0.001). The locomotion trajectories and behavioral changes of the shoaling assay can be found in Figure 8G,H and Supplementary Video S5.

### 2.5. C_60_ NPs Exposure Induces the Dysregulation of Circadian Rhythm

**Figure 9:**
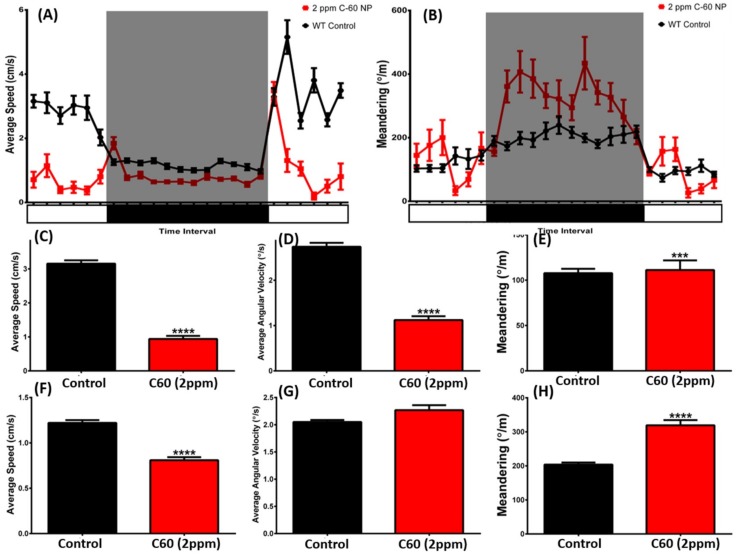
The circadian rhythm assay for wild-type and C_60_ NPs-exposed zebrafish after 12-day exposure. Comparison of the time chronological changes of the average speed (**A**) and meandering (**B**) between wild-type and C_60_ NPs-exposed fish in day and night cycle. The grey area shows the dark period and the unshaded area is the light period. Comparison of the average speed (**C**), average angular velocity (**D**), and meandering (**E**) at day cycle. Comparison of the average speed (**F**), average angular velocity (**G**), and meandering (**H**) at night cycle. The data are expressed as the mean ± SEM and were analyzed by Mann–Whitney test (*n* = 28 for control; *n* = 18 for C_60_ NPs-exposed fish; *** *p* < 0.005, ****, *p* < 0.0001).

For the circadian rhythm test, three behavioral endpoints were assessed in terms of average speed, average angular velocity, and meandering for each day and dark cycle. Time chronological test showed C_60_ NPs exposure could reduce the circadian locomotion activity (Figure 9A) and increase meandering (Figure 9B) in both day and night cycles. Quantitative comparison showed, in the light phase, the average speed (Figure 9C) and average angular velocity (Figure 9D) of the C_60_ NPs exposed fish were significantly reduced compared to the control group. However, in the night phase, only the average speed (Figure 9F) of the C_60_ NPs exposed fish was significantly reduced than the control group. Meandering is an index to show the total turning angles per meter swimming. Higher meandering means higher zig-zag swimming behavior. We found the meandering level was significantly higher for both day (Figure 9E) and night cycle (Figure 9H) in the C_60_ NPs exposed fish. The behavioral changes in the circadian test can be found in Supplementary Video S6.

### 2.6. C_60_ NPs Exposure Reduced the Color Preference Index

**Figure 10:**
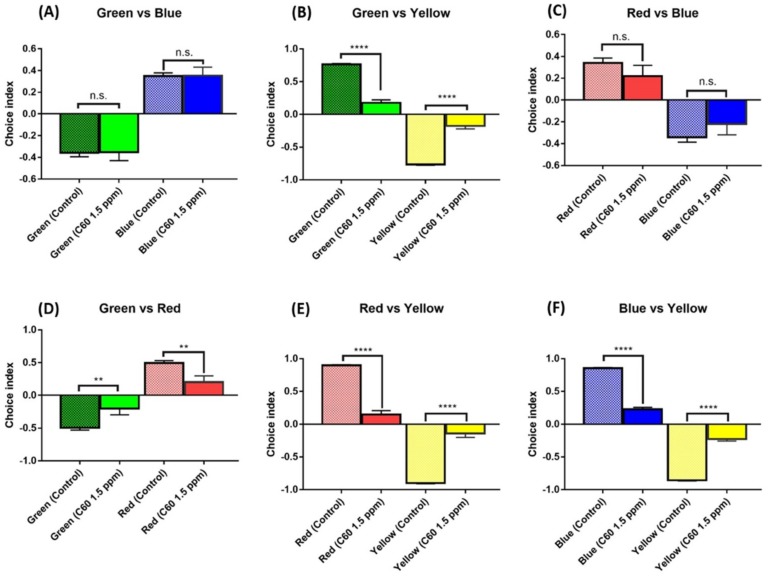
Comparison of color preferences between control and C_60_ NPs-exposed zebrafish after 12-day exposure. (**A**) Green vs. blue combination, (**B**) green vs. yellow combination, (**C**) red vs. blue combination, (**D**) green vs. red combination, (**E**) red vs. yellow combination, (**F**) blue vs. yellow combination. Data were analyzed using one-way ANOVA followed by the Tukey post-hoc test. If the data were not normally distributed, the data were analyzed using non-parametric Kruskal–Wallis followed by Dunn’s post-hoc test, and *p* < 0.05 were considered significantly different. The data are presented with mean ± SEM with *n* = 24, ** *p* < 0.01, **** *p* < 0.0001. n.s.: non-significance.

The C_60_ NPs-exposed fish displayed depression and social withdrawal-like behavior by showing lower aggression and loose shoaling. This interesting phenomenon led us to wonder whether chronic exposure to C_60_ NPs also reduce zebrafish’s general interest to other visually-based behaviors. We tested this hypothesis by performing a color preference test. For normal conditions, the wild-type fish displayed normal color preference in the following sequence of red > blue > green > yellow. After C_60_ NPs exposure, the color preference pattern did not change compared with the wild type for green/blue (Figure 10A; *p* > 0.999) and red/blue combinations (Figure 10C; *p* > 0.6057). However, their color preference was greatly reduced in green/yellow (Figure 10B; *p* < 0.0001), green/red (Figure 10D; *p* < 0.0087), red/yellow (Figure 10E; *p* < 0.0001), and blue/yellow (Figure 10F; *p* < 0.0001) combinations. The decrease in color preferences can be correlated with depression-like behavior which have been reported before [34].

### 2.7. Impact of C_60_ NPs on Oxidative Stress and Lipid Peroxidation Markers

Effects of sub-chronic exposure to C_60_ NPs on selected oxidative stress and lipid peroxidative markers are presented in Table 1. First, we measured the ROS levels (by measuring H_2_O_2_ concentration) and found this oxidative stress marker level was significantly elevated in both gill and muscle tissues in C_60_ NPs exposed fish. The notable elevation of oxidative stress in C_60_ NPs exposed fish led us to ask whether it caused any side effect on lipid peroxidation. We addressed this question by measuring three lipid peroxidation markers, namely, thiobarbituric acid reactive substances (TBARS), Malondialdehyde (MDA), and 4-hydroxy-2-nonenal (4-HNE). The activity of TBARS activity in muscle tissue was greater in groups treated with 2-ppm C_60_ NPs (Table 1; *p* < 0.001), but significant difference (Table 1; *p* < 0.05) was obtained in gills than in the control group. The activity of MDA was significantly lower (Table 1; *p* < 0.001) in gills of C_60_ NPs exposed zebrafish; on the contrary in muscle tissue the MDA activity show no difference between control and C_60_ NPs-exposed groups. However, the activity of 4-HNE was increased (Table 1; *p* < 0.01) in gills of the 1-ppm exposed C_60_ NPs group than in the control group. No significant difference between the experimental groups and the control in muscle tissues were observed in 4-HNE content.

### 2.8. Effect of C_60_ NPs on Antioxidant Enzymes

Next, we evaluated the anti-oxidative capacity in the gill and muscle tissues of C_60_ NPs exposed fish by measuring two markers of SOD (superoxidase dismutase) and catalase activities. Table 1 presents the significant changes in the activities of anti-oxidant enzymes in gill and muscle tissues of adult zebrafish. The catalase activity in the gill of adult zebrafish was significantly reduced (*p* < 0.001) in 2-ppm C_60_ NPs exposed groups as compared to the control. However, the catalase activity in muscle was slightly decreased (*p* < 0.05) in the treatment group than in the control zebrafish. The SOD activity in the gill of zebrafish was statistically significant lower (*p* < 0.05), even though the actual difference from the control group was minor. In the muscle, the SOD activity was decreased in 2-ppm C_60_ NPs (Figure 10B; *p* < 0.05). There was no significant difference in the 1-ppm C_60_ NPs exposed groups both in gill and muscle tissues. These results indicated that C_60_ NPs inhibited the production of antioxidant enzymes in adult zebrafishes.

### 2.9. Effect of C_60_ NPs on Stress and Inflammatory Markers

A slight increase in catecholamine content (*p* < 0.05) was found in fish exposed to 2 ppm of C_60_ NPs in gills compared to the control. Significantly higher (*p* < 0.001) catecholamine content in muscle tissues was found in fish exposed to 2 ppm of C_60_ NPs compared to the control animals. The present study also tested the inflammatory marker level in both gills and muscles of the experimental group and controls (Table 1). Cortisol level was also tested in zebrafish treated with C_60_ NPs. Compared to the controls, the TNFα level in groups exposed to 2 ppm of C_60_ NPs exhibited statistically significant (*p* < 0.01) increases in both gill and muscle tissues. The IL1β level in gill and muscle were greatly increased (*p* < 0.0001) compared to that of the control. In addition, the significantly higher single stranded DNA (ssDNA) level was found in fish exposed to 2 ppm of C_60_ NPs in muscle and gill groups (*p* < 0.001, *p* < 0.05) compared to the control group. The level of cortisol was significantly higher both in gill and muscle tissue for the 1-ppm and 2-ppm C_60_ NPs exposed group compared to the control (Table 1; *p* < 0.05, *p* < 0.01).

### 2.10. C_60_ NPs Induced Creatine Kinase Activity and Hypoxia Signaling

Creatine kinase is a key enzyme expressed in multiple tissue and plays a role to convert creatine to phosphocreatine (PCr) and ADP by utilizing the energy from ATP. In tissues that consume ATP rapidly, such as muscle and gill, PCr serves as an energy reservoir for the rapid buffering and regeneration of ATP in situ. Creatine kinase activity has been reported as a robust stress marker since its activity, both at mRNA and protein levels, showed overshooting when aquatic animals face environmental challenges [35]. In line with previous findings, we found a significant elevation of creatine kinase and reduction of ATP levels in both muscle and gill tissues of C_60_ NPs exposed zebrafish (Table 1). The sharp decline of ATP level in both muscle and gill supported the slow locomotion activity observed in C_60_ NPs exposed zebrafish. The activation of hypoxia signals decreases the cellular demand of ATP in cells and inhibits mitochondrial respiration [36]. Consistent with in vitro data, we found Hif1α, a key marker for hypoxia, was elevated in C_60_ NPs exposed zebrafish with low ATP levels in their muscle and gill tissues.

### 2.11. C_60_ NPs Induced Behavioral Abnormalities are Linked to Changes in Acetylcholine, Melatonin, and GABA Contents

**Figure A2:**
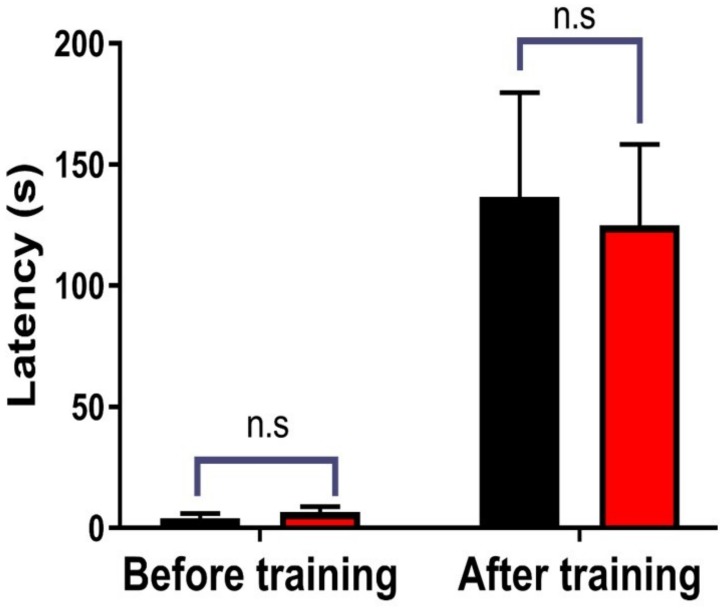
Passive avoidance test on training and memory of control (black) and C_60_ NPs (red) treated zebrafish after nine-day exposure. Data were presented as means ± standard error of the mean (SEM) (*n* = 12) and analyzed individually for each treated group. Statistical analysis was performed by one-way ANOVA, followed by Tukey post hoc test. n.s.: non-significance.

To examine the possibility of the loss of motor coordination (hypoactivity), anxiogenic behavior and circadian rhythm dysregulation induced by C_60_ NPs exposure, the protein expression levels of ACh (Acetylcholine), AChE (Acetylcholinesterase), dopamine, GABA (Gamma-aminobutyric acid), melatonin, and glutamate were measured by ELISA. Toxicity of C_60_ NPs in brain ACh and AChE activity has previously been reported in zebrafish [37]. Although we observed a significant increase in the brain AChE activity, inhibition in ACh level and reduction of glutamate level (Table 2), the short-term memory (tested by passive avoidance assay) for C_60_ NPs exposed zebrafish showed a similar level, compared to their control counterparts (Figure A2). Dopamine is a neurotransmitter secreted by the dopaminergic neurons of the midbrain and modulation of brain dopamine level is associated with anxiety-like behavior [38]. For C_60_ NPs exposed zebrafish, we found the brain dopamine level was three-fold elevated. GABA is an inhibitory neurotransmitter which plays a role for hypoactivity control by reducing neuronal excitability throughout the nervous system. By ELISA, we found the brain GABA level in C_60_ NP_S_ exposed fish was three-fold elevated (Table 2). This result suggests the C_60_ NP_S_ exposure might be damaging to the GABAergic neurons in zebrafish brain, thereby leading to hypoactivity behavior. In addition, melatonin, a key hormone on controlling circadian rhythm, showed great reduction in C_60_ NPs exposed fish. This result suggested that the C_60_ NP_S_ exposure might damage the melatonin producing cells in zebrafish brain, thus leading to circadian rhythm dysregulation.

## 3. Discussion

There is a growing concern for the potential toxicity of engineered nanomaterials that have made their way into virtually all novel applications in electronics, healthcare, cosmetics, technology, and engineering industries, in particular, biomedical products. Timely evaluation of nanomaterial toxicity will not only help regulatory agencies in assessing environmental and health risks of commercial nanomaterials but also provide industry with information to better direct the development of safer nanomaterials and products [39,40]. To date, there are some studies on long-term exposure to engineered nanoparticles, including fullerenes and fullerenes derivatives using adult zebrafish as whole animal-based testing assay to assess the potential toxicity of engineered nanomaterials [41,42,43,44]. In this study, a test panel consisted of multiple behavioral endpoints was employed to assess the potential toxicity of the long-term effects of C_60_ NPs in zebrafish. This panel of behavioral endpoint analyses included eight parameters: locomotion test, novel tank exploration, aggression, predator test, social interaction, shoaling, short-term memory, color preference, and circadian rhythm test. We evaluated the toxic effects of sub-chronic low concentrations of C_60_ NPs exposure on adult zebrafish and report for the first time that exposures to environmentally relevant levels of C_60_ NPs resulted in significant changes in neurobehavioral and toxicological effects in zebrafish. Sub-chronic (12 days) treatment with 2 ppm of C_60_ NPs produced the most significant effects on some of the tested parameters of behavioral analyses (locomotion, aggression, and circadian rhythm) as well as biochemical assay results.

The physicochemical properties of C_60_ NPs promotes the hypothesis that this nanoparticle might induce oxidative stress following photoactivation [45]. Carbon 60 has a unique spherical cage-like structure which can hold up to six electrons [46]. Through the electric dipole moment these electrons aligned around a ring of six carbon atoms. When C_60_ is acted upon by light, it emitted a higher energy level started producing a singlet of C_60_, which in turn reacts with O_2_ to form a singlet oxygen (^1^O_2_) [47]. Carbon 60 has an unique dual function; it can excite both the visible and ultraviolet light and then generate reactive oxygen species (ROS), specifically as superoxide and singlet oxygen [48]. These byproducts can induce oxidative stress promoting to various detrimental downstream effects such as DNA and protein adduction and lipid peroxidation/oxidation and cellular death [49,50].

Treatment with C_60_ NPs at concentrations of 2 mg/L produced the most significant effects on all the tested behavioral parameters (locomotion, exploratory, and aggression, etc.) than the 1 mg/L C_60_. These observations highlighted the significant role of nanoparticles in neurotoxic effects. Furthermore, C_60_ NPs at high concentrations decreased the degree of locomotion activity (hypoactivity) and the normal kinetic pattern of the zebrafish, showing behaviors similar to those observed in neurodegenerative diseases. In terms of exploratory behavior, C_60_ NPs significantly reduced the normal exploration behavior of adult fish. However, the aggression test also revealed the C_60_ NPs significantly reduced the aggressive nature of zebrafish, which plays a crucial role in the behavior and ecology of adult fish.

In vivo evaluations conducted using adult zebrafish provided strong evidence indicating that C_60_ NPs are capable of inducing toxicity in the tested fish. Four lines of evidence presented herein point to oxidative stress as a primary pathway of toxicity. The first line of evidence is that, when photoexcited, C_60_ NPs can induce membrane damage by generation of ROS in a concentration and time-dependent manner. Our biochemical assays revealed the ROS level was high in 2 ppm of C_60_ NPs treated adult fish. These findings are consistent with membrane damage in rats [51,52] and cell culture studies that found C_60_ induces cell death independent of apoptotic cell signaling [52]. A second line of evidence is given by the change in sensitivity of adult zebrafish to C_60_ NPs exposure that altered levels of antioxidants SOD and catalase. Superoxidase dismutase (SOD) is considered a first line defense mechanism against the deleterious effects of O_2_ radicals in cells, and it scavenges ROS by catalyzing the dismutation of SOD to H_2_O_2_ [53]. This enzyme is found in all living cells, and catalyzes the dismutation of superoxide into oxygen and hydrogen peroxide. In the present study, it was observed that there was a change in the activities of SOD and H_2_O_2_ at low concentrations of C_60_ NPs exposure. The lack of anti-inflammatory function observed from both gill and muscle tissues when fish were exposed to C_60_ NPs with elevated ssDNA and TNF-α level provided the third line of evidence suggesting an oxidative stress mechanism of toxicity. Yang and colleagues in 2012 demonstrated that C_60_ NPs caused DNA/RNA damage or other deleterious toxic effects at a systemic level and suggested one should be cautious to handle these NPs in numerous biomedical applications [54]. Similarly, our results displayed a significant increase in inflammatory biomarkers and ssDNA after the exposure of C_60_ NPs to zebrafish. The final line of evidence points to a hypoxia induced oxidative stress mechanism of C_60_ NPs toxicity. An increase in ROS induced hypoxia inducible factor 1α (Hif-1α) implicates an oxidative stress response and is in accordance with a previous report [55].

To understand the molecular mechanisms involved in behavioral impairments of C_60_ NPs exposed zebrafish, we analyzed the biochemical concentrations of various neurotransmitters in the brain. We found significant elevation of AChE levels and an inhibition in melatonin activity after the C_60_ NPs treatments to the zebrafish. There is evidence to suggest that AChE is an important regulator of apoptosis, which can be stimulated by a variety of apoptotic signaling and contributes to various physiological processes including cell proliferation and survival [56,57]. It is also a well-known fact that apoptosis is the fundamental element in neurotoxic effects of various compounds. Moreover, exposure of neurotoxic compounds such as aluminum [58] and ethanol [59] significantly elevated zebrafish brain AChE levels in previous reports. The results demonstrated in this study provide adult zebrafish exposed to C_60_ NPs have shown elevated AChE activity in brain and oxidative stress, and lipid peroxidation in muscle and gill tissues. Several reports show that cell membrane lipid peroxidation was considered as the key mechanism of toxicity caused by carbon fullerenes exposure [60,61].

## 4. Materials and Methods

### 4.1. C_60_ NPs Suspension

Carbon 60 NPs were purchased from Sigma–Aldrich (99.9% purity, catalog number 572500). Because of its insoluble nature in water, C_60_ NPs were sonicated overnight to make a uniform suspension in 1% Dimethyl Sulfoxide (DMSO) as described previously [15]. Then the solution was filtered through a 0.22 µm nylon Osmonics filter and the solutions were kept in dark. The stock solutions of 100 mg/L C_60_ NPs were prepared based on the maximum achievable concentration of nanoparticles to dissolve in DMSO. The highest exposure concentration was made by adding 0.01% of the DMSO-fullerene suspension to the fish water. The use of DMSO was suitable to increase the uptake of nanoparticles into the zebrafish. It is worth noting that the present study was specifically designed to assess the interactions between nanomaterials and the biological system, for instance an environmental exposure scenario.

### 4.2. Morphological and Structural Characterization

The morphology and structural properties C_60_ NPs were characterized by Scanning Electron Microscope (SEM) and X-ray Diffraction (XRD) methods. The morphology and individual diameter distributions of C_60_ NPs were confirmed by Field Emission Scanning Electron Microscope (JEOL-JSM-7600FESEM). The C_60_ NPs were suspended in 100 mg/L DMSO, stirred, and then sonicated overnight; pipetted 50-µL droplets were deposited on a copper grid and dried in a laboratory power controllable microwave oven for 4 h without vacuum treatment. The copper grids were directly inserted into the FESEM after they were completely dried. The images were taken at 10 K magnification by a dedicated CCD (charge-coupled device) camera. To reveal the crystallinity and phase of C_60_ NPs, we recorded the powder X-ray diffraction patterns in a RIGAKU diffractometer using Ni-filtered Cu-K radiation. (λ = 0.154 nm).

### 4.3. Animal Ethics

All the experimental protocols and procedures involving zebrafish were approved by the Committee for Animal Experimentation of the Chung Yuan Christian University (Number: CYCU104024, issue date 21 December 2015). All experiments were performed in accordance with the guidelines for laboratory animals.

### 4.4. Zebrafish Exposed to Fullerene Nanoparticle

The experimental design was depicted in Figure 1. For sub-chronic toxicity test, the control fish were not exposed to any nanoparticle or solvent, while zebrafish in the experimental group were treated with C_60_ NPs at different doses. About 70% of the water was changed every 24 h with redosing after each change. Two concentrations, 1 and 2 ppm, respectively, of C_60_ NPs were selected based on previous studies on other vertebrates [13,24,62]. Fish were fed two times a day on a diet of lab grown live brine shrimp.

### 4.5. Behavior Tests

The behavioral analysis was performed in the morning (9:00 to 12:00) and started with a 5-min pre-acclimation in the test tank. The endpoints were as follows: locomotion test (3D tracking), novel tank, mirror biting, predator avoidance, social interaction, shoaling, circadian rhythm assay, and color preference test. In addition, a memory test was also performed and the results showed no significant differences between the control and exposed group (in Supplementary Materials A2). A Canon EOS D600 camera was used to record the behavior of the zebrafish and idTracker software [25] was used for zebrafish locomotion tracking. After the behavioral tests, fish were anesthetized and immediately euthanized by immersion in tricaine (A5040, Sigma, St. Louis, MO, USA), and their gills and muscle tissues were removed for subsequent biochemical analyses.

A locomotion activity test was performed on the 5th day of the C_60_ NPs treatment. The locomotive activities of the zebrafish treated with C_60_ NPs were observed in a home-made apparatus, which consisted of a two chambered PP (polypropylene) box (20 × 20 × 20 cm). The tracking strategy for zebrafish 3D locomotion followed the protocol described in our previously published study [28]. The adult zebrafish after C_60_ NPs exposure at 2 ppm were used for 3D locomotion test. Three separate trails were performed using the same cohort of zebrafish and the same batch of C_60_ NPs.

Adult exploration behavior was evaluated on the 9th day of the C_60_ NPs exposure. The experiment was conducted in a temperature-controlled room (25 ± 1 °C) between 10:00 and 13:00 pm during the light phase. For novel tank exploration untreated adult zebrafish (*n* = 30) and carbon fullerene treated zebrafish (*n* = 30) were placed in the experimental tank filled with 1.25 L of fish water. The behavioral responses were recorded for 1 min at intervals of, 0, 5, 10, 15, 20, 25, and 30 min. The videos for each test were captured by a Canon EOS 600D camera with long range zoom lens. The behavioral endpoints for exploratory test were: time spent in the upper zone, freezing time movement, average speed, number of entries to the top, total distance travelled on top, maximum and minimum speed, swimming, and rapid time movement percentage. The videos were analyzed by using idTracker, and subsequently the tracking data were calculated using Microsoft Excel. The time spent on top duration indicated an anxiolytic behavior index of the C_60_ NPs exposed fish, on the contrary when the control zebrafish was introduced to a new environment, they spent more time at the bottom and gradually moved to the upper zone after few minutes [63].

To determine the zebrafish aggression, mirror biting test followed a protocol described in a previously published study [64] with some modifications. Control zebrafish (*n* = 30) and C_60_ NPs treated adult fishes (*n* = 30) were introduced into the experimental tank containing a mirror placed vertically to one side of the wall. Individual adult zebrafish were placed into the experimental tank and after 3 min of habituation, their behavior were recorded for 5 min. The aggressive behavior parameters were: average speed, mirror biting time percentage, longest duration on the mirror side, freezing time movement ratio, swimming, and rapid movement time. The mirror biting zone was set at the area 5 cm from mirror.

To capture the fear and escape behavior in zebrafish we used a predator (*Amatitlania nigrofasciata*) in the experimental tank. The predator avoidance test was performed in the experimental tank with a transparent glass separator placed at 15 cm away from the vertical side wall. Adult zebrafish, the untreated fish (*n* = 30) and C_60_ NPs treated fish (*n* = 30), were allowed to habituate to the experimental conditions for 5 min. After habituation period, the predator (convict cichlid *Amatitlania nigrofasciata* validated by 16S rRNA barcoding, body length 5 cm) was introduced into the other side of separator and the zebrafish behavior was recorded for 5 min. The fear response was determined by measuring the average speed, the distance travelled, the maximum and minimum speed, swimming, freezing, predator approaching time percentage, top/bottom ratio of time spent and travelling distance, and distance to predator separator in average.

The social interaction test was assessed on the 7th day of the C_60_ NPs treatment as previously described, with minor modifications [65]. In the social interaction test, untreated male zebrafish (*n* = 30) and C_60_ NPs treated zebrafish (*n* = 30) were placed in the tank filled with 1.25 L of water with a transparent glass separator placed at 11 cm away from the vertical side wall of the tank. The fish were allowed to habituate for 5 min. After habituation, another conspecific was placed on the other side of the separator to stimulate the social interaction behavior. The social response was determined by the following endpoints: Interaction time percentage, average speed, average distance to separator, average distance to separator.

A related social behavior, shoaling behavior, is common in many species of fish representing the complex interactions of animals moving together in coordinated movements. This behavior is related to foraging, mating, fear response, and defense against predators. In order to shoal fish must prefer to approach and remain near conspecifics. A shoal location task was conducted to quantify the shoal behavior. The shoaling test was performed on the 9th day of the C_60_ treatment and consisted of three fish for each shoal. A salient feature of zebrafish is its propensity to aggregate in groups and shows strong shoaling tendencies. After 5 min acclimation, average speed, time on top duration, average distance to center of the tank, inter fish distance, average nearest neighbor distance, and farthest neighbor distance were measured.

### 4.6. Circadian Rhythm Test

The sleep/wake behaviors were evaluated by circadian rhythm test on the 12th day of C_60_ NPs exposure. The test was based on previous publication with some modifications [66]. The light/dark cycle test apparatus consisted of 6 custom made small fish tanks (20 × 10 cm) which were placed above a light box. For the light cycle, a light emitting diode (LED) was used and to represent the dark cycle an infrared light emitting diodes (IR-LED) were used. An infrared camera with magnifying lens was located above the experimental setup to record the fish movements at 30 frames per seconds.

### 4.7. Video Tracking and Data Analysis

The tracked videos were recorded using an open source software, idTracker which converted the fish movement data to trajectories as previously described [28]. All tests were analyzed by Mann–Whitney *U* test followed by pairwise nonparametric analysis [67].

### 4.8. Total Protein Extraction from Tissues

After the behavioral analysis, three fishes were randomly collected from each tank (9 fish/treatment) for biochemical assays. Muscle and gill tissues were removed and a pool of three zebrafish tissues were used for homogenate preparation. Tissues were homogenized at medium speed with a Bullet blender (Next Advance, Inc., Troy, NY, USA) with 50 volumes of (v/w) ice cold phosphate saline buffer adjusted to pH 7.2. Samples were further centrifuged at 12,000× g or 15 min and the crude homogenates were stored in 100 µL aliquots at −80 °C until required. Tissue homogenates were also analyzed at the end of the behavioral experiment (day 12) to determine the possible effects of oxidative stress, lipid peroxidation, and antioxidant activity by the exposure of C_60_ NPs. Total protein concentration was determined using a Pierce BCA (bicinchonic acid) Protein Assay Kit (23225, Thermo Fisher Scientific, Waltham, MA, USA). The color formation was analyzed at 562 nm using a microplate reader (Multiskan GO, Thermo Fisher Scientific, Waltham, MA, USA).

### 4.9. Determination of Oxidative, Anti-Oxidative Capacity, Lipid Peroxidation, DNA Damage, Stress Hormone, and Inflammation Markers

The tissue oxidative stress marker of ROS (by measuring H_2_O_2_ level) was measured by using commercial target-specific ELISA kits (ZGB-E1561, Zgenebio Inc., Taipei, Taiwan). The tissue anti-oxidative capacity markers, catalase and SOD, were measured by using commercial target-specific ELISA kits (ZGB-E1598, ZGB-E1604, Zgenebio Inc., Taipei, Taiwan). The lipid peroxidation (4-hydroxy-2-nonenal, 4-HNE; Malondialdehyde, MDA and Thiobarbituric acid reactive substances, TBARS) and DNA damage (ssDNA) markers were measured by using commercial target-specific ELISA kits (ZGB-E1603, ZGB-E1592, ZGB-E1605 and ZGB-E1595, Zgenebio Inc., Taipei, Taiwan). Two stress hormones, catecholamine and cortisol, were measured by using commercial target-specific ELISA kits (ZGB-E1590, ZGB-E1560, Zgenebio Inc., Taipei, Taiwan). Two inflammation markers of TNF-α and IL-1β were measured by using commercial target-specific ELISA kits (ZGB-E1612, ZGB-E1608, Zgenebio Inc., Taipei, Taiwan). Initially, zebrafish tissues of gill and muscle were minced and completely homogenized in PBS solution by using tissue homogenizer. The target protein content of each sample was calibrated by interpolation from the standard calibration curve and normalized to the amount of total protein (μg) in each sample. The target protein content or activity was measured by following the manufacturer’s instructions. Some neurotransmitters such as GABA, melatonine, ACh, AChE, and glutamate were estimated from brain tissues of the C_60_ NPs exposed fish by using by using commercial target-specific ELISA kits (ZGB-E1574, ZGB-E1597, ZGB-E1585, ZGB-E1637 and ZGB-E1588, Zgenebio Inc., Taipei, Taiwan). The target-specific ELISA kits used in this study was based on Sandwich ELISA principle. First, the target-specific antibodies were immobilized onto 96-well microplates. Later the tissue homogenates and HRP (horseradish peroxidase)-conjugated target-specific antibodies were applied onto microplate and incubated at 37 °C for 1 h. After wash with washing buffer, chromogen A and B were applied onto microplate and incubated at 37 °C for 15 min. Finally, stop solution was applied to stop color development and the absorbance was analyzed at 450 nm using a microplate reader (Multiskan GO, Thermo Fisher Scientific, Waltham, MA, USA). The relative concentration of target protein was then quantified by comparing to the standard curve generated from the standard provided by commercial kits.

### 4.10. Statistical Analysis

The biochemical data were analyzed by individual fish: *n* = 9 for both control and C_60_ NPs exposed fish. All statistical analyses were plotted and compiled by using GraphPad prism (GraphPad Software version 7 Inc., La Jolla, CA, USA). Each fish group was compared using one-way ANOVA test followed by the post hoc test of Tukey, depending upon the data normality for significant data. Significant difference between control and treated groups was set at a *p* value < 0.005.

## 5. Summary and Conclusions

The present study provided direct evidence showing that the chronic exposure of C_60_ NPs induced multiple behavioral abnormalities in adult zebrafish. We have provided, for the first time, a detailed overview of behavioral changes associated with biological network disruptions upon exposure to engineered C_60_ NPs. Our results support the hypothesis that sub-chronic exposure to environmentally relevant concentration of C_60_ NPs (1 and 2 ppm) cause behavioral abnormalities related to hypoactivity, anxiety-like behavior, reduced aggression/fear, and circadian rhythm dysregulation in adult zebrafish. The method for neurotoxicity assessment based on behavioral analysis specifically light/dark cycle and locomotion introduced in the present study could be used as a rapid, comprehensive, and inexpensive method to identify the potential toxicity of various engineered nanomaterials. The presence of pathologies associated with lipid peroxidation in the gill and muscle, and the elevated ROS and TBARS levels in tissues suggest that the fish were suffering from overt oxidative stress. Our data also highlights some modes of C_60_ NPs toxicity that have not been identified in zebrafish before and require further investigation to elucidate the mechanisms. Biochemical results of the brain and muscle raises new concerns about neurotoxic effects of C_60_ NPs that may alter fish behaviors. Our dynamic adult zebrafish animal assay result can be used to reveal potential toxicity of newly engineered nanomaterials at cellular, physiological, and behavioral levels. Timely evaluation of nanomaterial toxicity will not only help regulatory agencies in assessing environmental and health risks of commercial nanomaterials, but also provide industry with information to better direct the development of safer nanomaterials and products.

## Figures and Tables

**Table 1:** Detection of biochemical parameters in the muscle and gill tissue for C_60_ NPs-exposed zebrafish after 12-day exposure.

Biomarker	Control	C60 (1ppm)	C60 (2ppm)	Unit	Significance	ANOVA F value	*p* value
**Muscle**							
ROS	4.643 ± 0.356 ^a^	5.353 ± 0.586 ^a^	15.710 ± 1.881 ^b^	U/μg total protein	YES	F (2, 6) = 28.740	*p* = 0.0008
4-HNE	1.028 ± 0.101	1.192 ± 0.223	1.005 ± 0.182	U/μg total protein	NO	F (2, 6) = 0.341	*p* = 0.7272
MDA	0.163 ± 0.011	0.096 ± 0.016	0.143 ± 0.021	ng/μg total protein	NO	F (2, 6) = 4.416	*p* = 0.0662
TBARS	3.317 ± 0.045 ^a^	3.922 ± 0.541 ^a^	20.240 ± 2.066 ^b^	ng/μg total protein	YES	F (2, 6) = 60.600	*p* = 0.0001
catalase	1.157 ± 0.003 ^a^	1.662 ± 0.200 ^a^	0.499 ± 0.045 ^b^	U/μg total protein	YES	F (2, 6) = 24.300	*p* = 0.0013
SOD	3.443 ± 0.199 ^a^	4.825 ± 0.787 ^a^	1.004 ± 0.204 ^b^	U/μg total protein	YES	F (2, 6) = 16.050	*p* = 0.0039
ssDNA	0.717 ± 0.007 ^a^	0.791 ± 0.162 ^a^	3.702 ± 0.229 ^b^	U/μg total protein	YES	F (2, 6) = 110.600	*p* < 0.0001
catecholamine	4.031 ± 0.063 ^a^	3.793 ± 0.732 ^a^	15.240 ± 1.847 ^b^	ng/μg total protein	YES	F (2, 6) = 32.480	*p* = 0.0006
cortisol	11.700 ± 0.413 ^a^	35.090 ± 5.455 ^b^	43.020 ± 5.051 ^b^	pg/μg total protein	YES	F (2, 6) = 14.340	*p* = 0.0052
TNF-α	5.877 ± 0.076 ^a^	5.603 ± 1.080 ^a^	19.880 ± 2.414 ^b^	pg/μg total protein	YES	F (2, 6) = 28.600	*p* = 0.0009
IL1β	0.406 ± 0.010 ^a^	0.365 ± 0.071 ^a^	2.651 ± 0.242 ^b^	ng/μg total protein	YES	F (2, 6) = 80.890	*p* < 0.0001
Hif-1α	13.880 ± 0.738	21.880 ± 4.270	19.170 ± 2.221	pg/μg total protein	NO	F (2, 6) = 2.095	*p* = 0.2042
creatine kinase	2.092 ± 0.019 ^a^	2.037 ± 0.412 ^a^	5.300 ± 0.473 ^b^	pg/μg total protein	YES	F (2, 6) = 26.600	*p* = 0.0010
ATP	293.400 ± 8.753 ^a^	257.900 ± 57.280 ^a^	89.440 ± 15.460 ^b^	ng/μg total protein	YES	F (2, 6) = 9.909	*p* = 0.0126
creatinine	5.715 ± 0.133	3.916 ± 1.222	5.543 ± 0.435	U/μg total protein	NO	F (2, 6) = 1.738	*p* = 0.2538
metallothionine	13.840 ± 0.537	13.470 ± 0.727	12.610 ± 0.564	pg/μg total protein	NO	F (2, 6) = 1.055	*p* = 0.4049
**Gill**							
ROS	2.923 ± 0.193 ^a^	3.386 ± 0.644 ^a^	7.235 ± 1.219 ^b^	U/μg total protein	YES	F (2, 6) = 8.675	P = 0.0170
4-HNE	0.552 ± 0.115 ^a^	1.048 ± 0.030 ^b^	0.792 ± 0.070 ^a,b^	U/μg total protein	YES	F (2, 6) = 9.693	*p* = 0.0132
MDA	0.123 ± 0.007 ^a^	0.114 ± 0.012 ^a^	0.024 ± 0.006 ^b^	ng/μg total protein	YES	F (2, 6) = 39.050	*p* = 0.0004
TBARS	2.504 ± 0.074 ^a^	3.135 ± 0.534 ^a^	6.630 ± 1.230 ^b^	ng/μg total protein	YES	F (2, 6) = 8.213	*p* = 0.0191
Catalase	0.875 ± 0.044 ^a^	0.815 ± 0.052 ^a^	0.481 ± 0.022 ^b^	U/μg total protein	YES	F (2, 6) = 26.470	*p* = 0.0011
SOD	2.501 ± 0.071 ^a^	2.946 ± 0.473 ^a^	1.088 ± 0.082 ^b^	U/μg total protein	YES	F (2, 6) = 11.990	*p* = 0.0080
ssDNA	0.522 ± 0.018 ^a^	0.941 ± 0.056 ^a^	1.555 ± 0.204 ^b^	U/μg total protein	YES	F (2, 6) = 17.930	*p* = 0.0029
catecholamine	3.088 ± 0.087 ^a^	5.676 ± 0.349 ^a, c^	10.430 ± 2.156 ^b, c^	ng/μg total protein	YES	F (2, 6) = 8.717	*p* = 0.0168
cortisol	6.024 ± 0.451 ^a^	11.830 ± 1.165 ^b^	13.010 ± 1.320 ^b^	pg/μg total protein	YES	F (2, 6) = 12.710	*p* = 0.0070
TNF-α	4.404 ± 0.138 ^a^	8.020 ± 0.482 ^a^	12.220 ± 1.465 ^b^	pg/μg total protein	YES	F (2, 6) = 19.150	*p* = 0.0025
IL1β	0.334 ± 0.007 ^a^	0.631 ± 0.045 ^a^	1.302 ± 0.117 ^b^	ng/μg total protein	YES	F (2, 6) = 46.850	*p* = 0.0002
Hif-1α	9.727 ± 1.484 ^a^	22.580 ± 3.800 ^b^	13.490 ± 2.089 ^a,b^	pg/μg total protein	YES	F (2, 6) = 6.240	*p* = 0.0342
creatine kinase	1.496 ± 0.091 ^a^	2.820 ± 0.168 ^b^	2.085 ± 0.243 ^a,b^	pg/μg total protein	YES	F (2, 6) = 13.810	*p* = 0.0057
ATP	142.700 ± 15.940 ^a^	208.400 ± 20.080 ^a,b^	112.300 ± 12.880 ^a,c^	ng/μg total protein	YES	F (2, 6) = 8.790	*p* = 0.0165
creatinine	5.035 ± 1.256	4.843 ± 0.768	5.036 ± 0.850	U/μg total protein	NO	F (2, 6) = 0.013	*p* = 0.9873
metallothionine	9.871 ± 0.340	12.770 ± 1.227	11.950 ± 0.820	pg/μg total protein	NO	F (2, 6) = 2.927	*p* = 0.1297

Enzymatic or ELISA (enzyme-linked immunosorbent assay)-based methods were applied to detect the ROS (reactive oxygen species), TBARS (thiobarbituric acid reactive substances), SOD (superoxidase dismutase), catalase, ssDNA (single-stranded DNA), catecholamine, cortisol, TNF-α (tumor necrosis factor alpha), IL-1β (interleukin 1 beta), Hif-1α, creatine kinase, ATP (adenosine triphosphate), creatinine, and metallothionein in muscle and gill for C_60_ NPs-exposed zebrafish, respectively. Data were presented as means ± SEM with three to six independent experiments. Statistical analysis was by one-way ANOVA test followed by a Tukey post-hoc test. Different labels above columns indicate a significant difference between different experimental groups with *p* < 0.01.

**Table 2:** Detection of biochemical parameters in the brain tissue for C_60_ NPs-exposed zebrafish after 12-day exposure.

Biomarker	Control	C60 (1ppm)	C60 (2ppm)	Unit	Significance	ANOVA F value	*p* value
ACh	22.750 ± 0.373 ^a^	15.520 ± 1.686 ^b^	9.309 ± 0.641 ^c^	U/μg totalprotein	YES	F (2, 6) = 39.99	*p* = 0.0003
AChE	3.102 ± 0.079 ^a^	3.272 ± 0.504 ^a^	7.527 ± 0.284 ^b^	U/μg totalprotein	YES	F (2, 6) = 55.34	*p* = 0.0001
Dopamine	11.860 ± 2.769 ^a^	11.940 ± 1.420 ^a^	34.530 ± 5.335 ^b^	pg/μg totalprotein	YES	F (2, 6) = 13.42	*p* = 0.0061
GABA	0.057 ± 0.003 ^a^	0.057 ± 0.001 ^a^	0.159 ± 0.013 ^b^	ug/μg totalprotein	YES	F (2, 6) = 59.43	*p* = 0.0001
melatonin	3.459 ± 0.143 ^a^	1.759 ± 0.035 ^b^	2.493 ± 0.301 ^b^	pg/μg totalprotein	YES	F (2, 6) = 19.43	*p* = 0.0024
glutamate	0.633 ± 0.069 ^a^	0.392 ± 0.024 ^a,b^	0.379 ± 0.055 ^b^	ug/μg totalprotein	YES	F (2, 6) = 7.312	*p* = 0.0246

Enzymatic or ELISA-based methods were applied to detect the ACh (acetylcholine), AChE (acetylcholinesterase), dopamine, GABA (gamma-aminobutyric acid), melatonin, and glutamate. Data were presented as means ± S.E.M with three to six independent experiments. Statistical analysis was by one-way ANOVA test followed by the Tukey post-hoc test. Different labels above columns indicate a significant difference between different experimental groups with *p* < 0.01.

## Supplementary Materials

Supplementary materials can be found at https://zenodo.org/record/1998318#.XAkl_LgRWUk. Video S1. Locomotion of wild type (left) and 2 ppm C_60_ NPs exposed (right) zebrafish in novel tank test. Locomotion was recorded and analyzed by using idTracker software. The upper chamber space was highlighted in yellow color. This video was played at 5× speed. Video S2. Locomotion of wild type (left) and 2 ppm C_60_ NPs exposed (right) zebrafish in the mirror biting test. The mirror was positioned to the right side of the wall. The mirror approaching zones is highlighted in yellow color. This video is played at 10× speed. Video S3. Locomotion of wild type (left) and 2 ppm C_60_ NPs exposed (right) zebrafish in the predator avoidance test. The predator was placed in the left side and zebrafish is placed in the right side. A transparent glass separator was placed at 15 cm away from the vertical side wall to separate zebrafish and the predator. The predator approaching zones is highlighted in yellow color. This video is played at 10× speed. Video S4. Locomotion of wild type (left) and 2 ppm C_60_ NPs exposed (right) zebrafish in social interaction test. The normal zebrafish is placed in the left side and tested KO fish is placed in the right site and one transparent glass plate is inserted to separate both animals. The social interaction approaching zones are highlighted in yellow color. This video is played at 10× speed. Video S5. Locomotion of three wild type (left) and 2 ppm C_60_ NPs exposed (right) zebrafish in shoaling test. This video was played at 10× speed. Table S1: Comparison of toxicity and potential effects of C60 in different model system.
